# Efficacy of Motor Imagery in the Treatment of Poststroke Dysphagia: A Systematic Review and Meta‐Analysis

**DOI:** 10.1002/brb3.70826

**Published:** 2025-09-02

**Authors:** Yunlu Liu, Yang Wang, Xinyu Zhao, Peiqun Hu, Yushi Hu

**Affiliations:** ^1^ Sports Medicine Key Laboratory of Sichuan Province, Key Laboratory of Sports Medicine, General Administration of Sport of China, School of Sports Medicine and Health Chengdu Sport University Chengdu China; ^2^ Postdoctoral Workstation Affiliated Sport Hospital of Chengdu Sport University Chengdu China; ^3^ School of Acupuncture and Tuina Chengdu University of Traditional Chinese Medicine Chengdu China

**Keywords:** efficacy, meta‐analysis, motor imagery, poststroke dysphagia

## Abstract

**Background:**

Poststroke dysphagia impairs patients' quality of life and survival. Motor imagery (MI) is increasingly used as a rehabilitation adjunct, but its efficacy requires validation. This study aims to evaluate MI's effectiveness in treating poststroke dysphagia.

**Methods:**

An extensive literature search was conducted across eight English and Chinese electronic databases (from inception to April 2025) to identify randomized controlled trials (RCTs) comparing the effects of MI combined with conventional therapy (experimental group) versus conventional therapy alone (control group) on poststroke dysphagia. The methodological rigor was assessed using the Cochrane Collaboration Risk of Bias Tool. Data analysis of the outcome measures was conducted using RevMan 5.3 software, with bootstrapped analysis to test the accuracy of primary outcomes.

**Results:**

Pooled analysis of 13 RCTs involving 1053 patients with poststroke dysphagia demonstrated that, compared to the control group, the MI supplementation group showed significantly lower water swallowing test scores (MD: −0.64, 95% CI: −0.76, −0.51, *p* < 0.00001) and standardized swallowing assessment scores (MD: −2.26, 95% CI: −2.94, −1.57, *p* < 0.00001) and higher swallowing quality of life scores (MD: 22.03, 95% CI: 7.25, 36.81, *p* = 0.003). Bootstrapping analysis substantiated the precision of these results. Furthermore, the meta‐analysis revealed that add‐on MI therapy significantly reduced the incidence of aspiration pneumonia (RR: 0.25, 95% CI: 0.12, 0.53, *p* = 0.0003) and increased clinical efficacy (RR: 1.23, 95% CI: 1.08, 1.39, *p* = 0.001). However, no significant effects were observed on aspiration incidence, malnutrition, or nasogastric tube removal rates.

**Conclusion:**

Our findings support that MI therapy can be implemented as an add‐on approach for poststroke dysphagia. More high‐quality RCTs from multicenters are needed to provide more reliable evidence and explore the optimal treatment protocol.

## Graphical abstract

This systematic review and meta‐analysis synthesized data from 13 studies (*n *= 1053) assessing motor imagery (MI) for poststroke dysphagia. We provide the first meta‐analytic evidence that MI as an add‐on intervention benefits dysphagia management poststroke. Further high‐quality, large‐scale multicenter RCTs are needed to strengthen evidence for clinical application of MI.

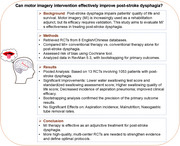

## 1 Introduction

Stroke, or cerebrovascular accident, is characterized by a sudden neurological impairment caused by an unexpected disruption of blood flow to the brain due to either occlusion or hemorrhage (Woods 2023). According to the Global Burden of Disease (GBD), stroke is the second leading cause of death, resulting in approximately 7 million fatalities each year. Among survivors, about 36%–71% will suffer from varying degrees of disability, and the impact can be devastating, affecting physical, cognitive, and mental functions (Feigin et al. 2025; Yang et al. 2016). As the primary contributor to global neurological disease burden, the annual cost of productivity loss following stroke was estimated to be $38.1 billion and €12 billion in the US and Europe, respectively (Luengo‐Fernandez et al. 2020; Strilciuc et al. 2021). Obviously, the stroke‐related burden poses a difficult challenge to society universally, and effective approaches should be taken to reduce the global stroke burden.

Dysphagia is the most prevalent disability resulting from stroke, often occurring early and persisting for 6 months post‐onset (Cohen et al. 2016). Poststroke dysphagia not only compromises quality of life but can also result in severe complications, including aspiration pneumonia, malnutrition, dehydration, and so forth. These serious complications of dysphagia are strongly linked to mortality and disability (Paciaroni et al. 2004; Rofes et al. 2011). Physiologically, swallowing movements are regulated by local reflexes in the esophagus and pharynx, brainstem feedback, and the cortical motor area's swallowing center (Miller 2008). Unilateral and bilateral lesions of the cerebral cortex, as well as brainstem lesions, may impair normal swallowing function, specifically, interrupting volitional control of chewing and the bolus transportation process, thereby leading to dysphagia in stroke patients (Daniels et al. 1999).

For many years, rehabilitation management for poststroke dysphagia included dietary interventions, nutritional interventions, and behavioral strategies (Hadde and Chen 2021). However, these approaches are either associated with potential adverse effects of sarcopenia or should be carefully carried out on a case‐by‐case basis (Cohen et al. 2016). Currently, neurostimulation techniques showing promise in modulating neuroplasticity of the swallowing network have been developed with the aim of helping stroke patients regain normal swallow function (Cabral et al. 2022). But the effect of neurostimulation techniques on important outcome indicators, such as aspiration pneumonia and mortality, needs further confirmation. Moreover, as swallowing is a complex and multifactorial physiological process that requires orchestration of neural mechanisms distributed across the peripheral and central nervous systems, the use of an inappropriate paradigm of stimulation therapy could eventually lead to maladaptive neural plasticity, finally hindering swallowing recovery (Martin 2009).

Recently, motor imagery (MI) has been applied in the treatment of poststroke dysfunction, alongside conventional strategies to promote functional recovery (Zimmermann‐Schlatter et al. 2008). Initially, MI was developed to help elite athletes to promote their sports performance (Guillot and Collet 2008). With the advancements in dynamic electroencephalography and functional magnetic resonance imaging (fMRI), the neural representations of imagined movements have been shown to be similar to those of actual motor execution (Munzert et al. 2009). This realization has led to the growing incorporation of MI into neurological rehabilitation practices. Generally, MI is seen as a mental rehearsal where an individual mentally rehearses an action without any physical movement or even muscle tensing (Mulder 2007). Accumulating evidence has shown that MI is not only beneficial for poststroke functional recovery but also highly accessible for patient self‐administration in daily practice (Guerra et al. 2017; Lambert et al. 2023; R.‐Q. Li et al. 2017; Yan et al. 2024). The underlying mechanism of MI intervention involves the emulation theory of representation. As the most widely accepted theory, it posits that during MI, the brain's control center engages an “emulator” to simulate the execution of physical movements while bypassing the brain's usual pathways that regulate muscle activity. This “emulator” replicates the neural circuits established by the brain, serving to enhance the sensory feedback loop and mitigate the impact of feedback delay issues. MI training involves the continuous offline activation of the “emulator” through efferent neural currents, which facilitates the repair or reorganization of impaired motor pathways, boosts synaptic efficiency, and reduces presynaptic inhibition (Grush 2004; F. Li et al. 2018; Ruffino et al. 2017). Overall, MI constitutes a mental execution integrating sensory, motor, and cognitive processes.

Various randomized controlled trials (RCTs) have demonstrated that MI can be used as a supplementary method to conventional therapy in facilitating the rehabilitation of poststroke dysphagia patients (Cao et al. 2014; G. Li et al. 2020; Xu 2021). However, no meta‐analysis has focused on the effect of MI on swallowing function. Therefore, with the research question as follows: “Can MI intervention effectively improve poststroke dysphagia?”, the present study aimed to systematically assess the efficacy of MI on swallowing function in stroke patients. We hope this meticulously conducted meta‐analysis and systematic review will furnish robust evidence to support the clinical utilization of MI in facilitating recovery from poststroke dysphagia.

## 2 Material and Methods

This meta‐analysis follows the Preferred Reporting Items for Systematic Reviews and Meta‐Analyses (PRISMA) guidelines and is registered on PROSPERO with the registration number CRD42024575810.

### 2.1 Search Strategy

Both English and Chinese databases have been searched comprehensively, such as Web of Science, PubMed, Cochrane Library, EMBASE, China National Knowledge Infrastructure (CNKI), Sinomed, Chinese Scientific Journal Database (VIP), and WANFANG databases. The search period is from database inception to April 2025. The following terms and combinations were utilized in the extensive search for the potential eligible literatures: [“imagery, psychotherapy” (MeSH Terms) OR “motor imagery” OR “imagery” OR “mental imagery” OR “mental practice” OR “mental training” OR “mental rehearsal”] AND [“stroke” (MeSH Terms) OR “Hemorrhagic Stroke” (MeSH Terms) OR “Ischemic Stroke” (MeSH Terms)] AND [“Deglutition Disorders” (MeSH Terms) OR “swallowing dysfunction” OR “impaired swallowing”]. Potential articles in the citation lists were also screened to further search for validation.

### 2.2 Inclusion and Exclusion Criteria

Included studies met the following criteria: (1) patients with hemorrhagic stroke or ischemic stroke. (2) RCT. (3) MI was applied in combination with a conventional therapy in the experimental group, while the control group only received the same conventional therapy used in the experimental group. (4) At least one of the outcome measurements was documented in the RCT. (5) There were no restrictions regarding age, gender, disease stage, duration, and frequency of treatment.

The exclusion criteria were as follows: (1) non‐clinical RCTs. (2) Patients participating in the study without a confirmed diagnosis of stroke. (3) Literature lacking essential data. (4) Duplicate studies. (5) Patients in the trial group received a combination of MI and additional treatment strategies alongside the conventional therapy provided to the control group.

### 2.3 Outcome Measures

In this study, the primary outcome measures were as follows: water swallowing test (WST), standardized swallowing assessment (SSA), and swallowing quality of life (SWAL‐QOL). The secondary outcome measures of this study included clinical efficacy, complications, and nasogastric tube removal rate.

### 2.4 Study Selection and Data Extraction

A systematic search was conducted on the aforementioned electronic bibliographic databases following a predefined strategy. First, duplicate records were removed. Then, two reviewers independently screened the titles and abstracts of the articles to identify studies that fulfilled the predefined eligibility criteria. The full texts of these eligible studies were obtained for further detailed assessment. A pilot phase with three articles was carried out to create a standardized data extraction template that included various sections: study characteristics (such as title, lead author, language, publication, and year), participant demographics (including age, gender distribution, total number of participants, course, and type of disease), intervention details (including treatment strategies, duration, and frequency in experimental and control groups), and outcome measures. Excluded studies were documented along with the reasons for their exclusion. Two reviewers extracted data from the full texts, resolving discrepancies through discussion or consulting a third one if needed.

### 2.5 Methodological Quality Assessment

Utilizing the Cochrane Collaboration's Risk of Bias Tool (Higgins et al. 2011), two researchers assessed the methodological quality of the included studies independently. The assessment covered seven key domains: (1) random sequence generation, (2) allocation concealment, (3) blinding of participants and personnel, (4) blinding of outcome assessment, (5) incomplete outcome data, (6) selective reporting, and (7) other bias. The risk of bias for each domain was categorized as low, high, or unclear. Any disagreements were resolved through consensus discussions, ensuring that a unanimous decision was reached in all cases.

### 2.6 Statistical Analysis

For continuous outcomes, the mean difference (MD) served as the measure of effect; for dichotomous variables, the relative risk (RR) was the measure of effect. The 95% confidence interval (CI) was applied to assess the overall effects. Heterogeneity was evaluated using the *I* ^2^ statistic. Values below 50% indicated no significant heterogeneity, so a fixed‐effect model was adopted for the meta‐analysis. When significant heterogeneity was detected (*I* ^2^ ≥ 50%), a random‐effects model was applied instead. Sensitivity analysis was conducted using a stepwise elimination approach to explore potential sources of heterogeneity. Subgroup analysis for an in‐depth assessment on the subject of the guided MI modality was conducted. Publication bias analysis, typically less critical with fewer than 10 studies, was planned to be conducted using funnel plots only when there were 10 or more studies reporting on a single outcome measure (N. Zhang et al. 2024). Review Manager 5.3 was used for performing meta‐analysis, whereas the bootstrapped meta‐analysis was conducted using the OpenMEE software (Wallace et al. 2017).

## 3 Results

### 3.1 Study Selection

**FIGURE 1:**
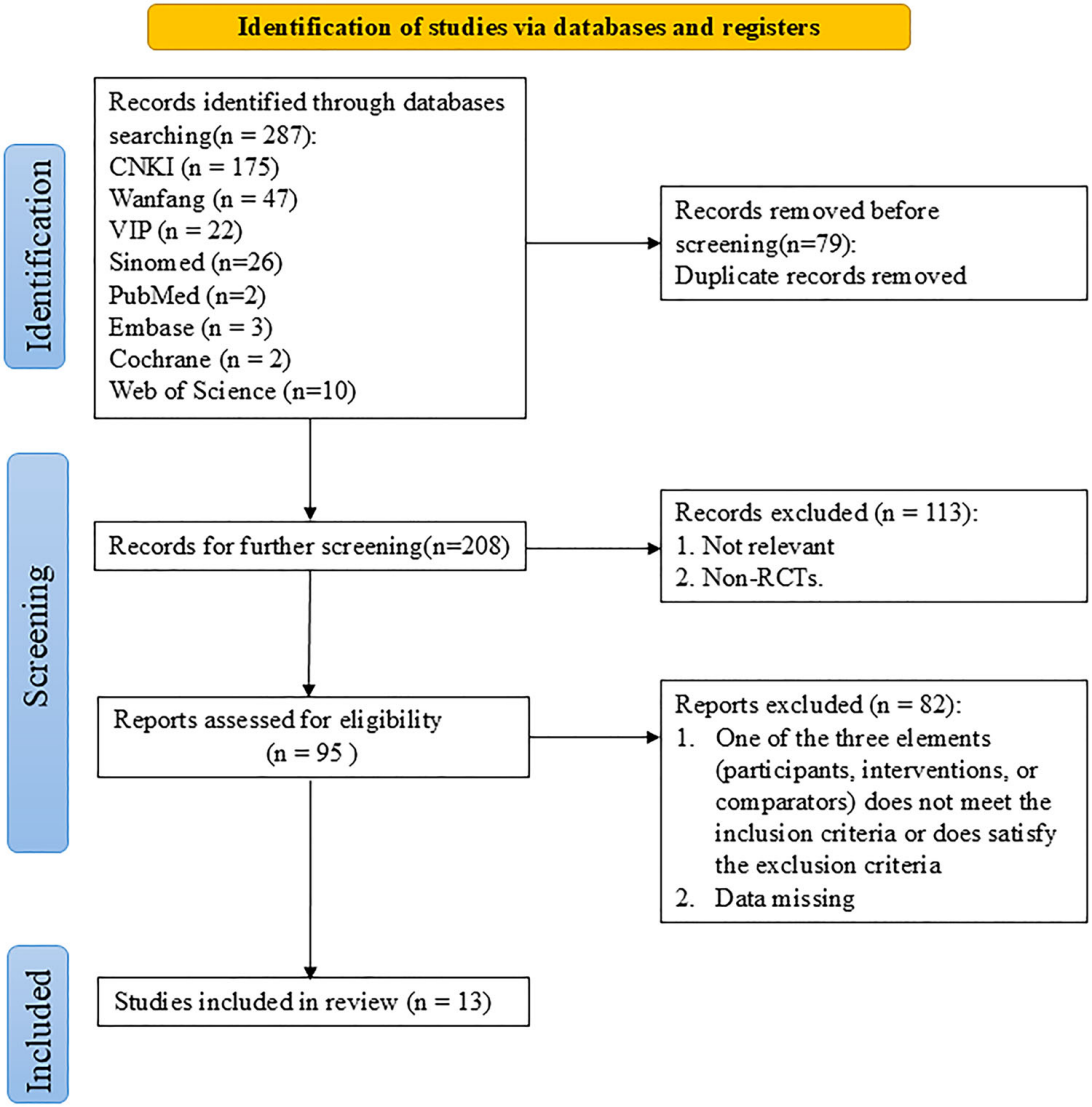
Flowchart of study selection.

A PRISMA‐compliant flow diagram representing the literature screening procedure is shown in Figure 1. A total of 287 articles were initially gathered from databases following the search strategy. The remaining 208 articles were identified after removing the 79 duplicates. By screening the titles and abstracts, we deleted 113 irrelevant and non‐RCT articles. After reviewing the full text of the remaining 95 articles, a total of 82 articles were excluded for not meeting the inclusion criteria or for meeting the exclusion criteria. Ultimately, 13 eligible RCTs were included in the meta‐analysis (Cao et al. 2014; Chen 2021; Deng 2017; G. Li et al. 2020; J. Liu et al. 2018; Luo et al. 2020; Xu 2021; Xu et al. 2017; Xu et al. 2018; Xu et al. 2019; Yang et al. 2023; H. Zhang and Ma 2017; Zhou 2020).

### 3.2 Study Characteristics

A total of 1053 patients were included, consisting of 529 in the experimental group and 524 in the control group. All 13 included studies were conducted in mainland China. All the patients presented hemorrhagic or ischemic stroke when reported in the included articles. The sample sizes in each group of included studies ranged from 27 to 103. The intervention in the control group was conventional treatment (the detailed conventional treatment of each included study is shown in Table S1), while that in the experimental group was conventional treatment combined with guided MI, which was performed by listening to swallowing instructions or by a combination of listening to auditory instructions and watching deglutition‐related actions. A total of six studies reported WST (Cao et al. 2014; Deng 2017; G. Li et al. 2020; Luo et al. 2020; Xu et al. 2018; Yang et al. 2023). Six studies showed the SSA (Chen 2021; J. Liu et al. 2018; Xu 2021; Xu et al. 2017; Xu et al. 2019; Zhou 2020). Five studies reported SWAL‐QOL (Chen 2021; J. Liu et al. 2018; Xu et al. 2017; Xu et al. 2019; Yang et al. 2023). Three studies reported the clinical effectiveness rate (G. Li et al. 2020; Xu et al. 2018; H. Zhang and Ma 2017). Five studies reported complications (Cao et al. 2014; Chen 2021; Deng 2017; G. Li et al. 2020; Xu et al. 2019). Two studies mentioned nasogastric tube removal rate (Cao et al. 2014; G. Li et al. 2020). Table 1 provides detailed characteristics of the 13 included studies. (The DOI or URL address of included studies is provided in Table S2).

**TABLE 1:** Characteristics of included studies.

Study ID	Group	Number (M/F)	Age, years	Type of stroke	Intervention and treatment duration	MI modality	Outcomes
G. Li et al. (2020)	Trial	30 (17/13)	64.34 ± 5.76	NR	CT + MI, 30 min per session, once daily, five sessions per week, 2 weeks constituting one course, a total of four courses	Guided by listening	①④⑤⑥
	Control	30 (16/14)	64.67 ± 5.68		CT		
Xu et al. (2018)	Trial	35 (20/15)	54.57 ± 3.54	Hemorrhage, infarction	CT + MI, 12–18 min per session, once daily	Guided by listening	①④
	Control	30 (17/13)	55.48 ± 3.27		CT		
Luo et al. (2020)	Trial	30 (19/11)	65.5 ± 14.5	NR	CT + MI, 15 min per session, once daily, 21 days constituting one course, a total of one course	Guided by listening	①
	Control	30 (18/12)	65.6 ± 19.4		CT		
Chen (2021)	Trial	50 (32/18)	53.6 ± 9.3	Hemorrhage	CT + MI, 20 min per session, once daily, for a total of 4 weeks	Guided by listening	②③⑤
	Control	50 (31/19)	53.4 ± 9.1		CT		
Xu et al. (2019)	Trial	30 (17/13)	63.14 ± 7.96	Hemorrhage, Infarction	CT + MI, 30 min per session, three sessions per day, a total of 6 weeks	Guided by listening	②③⑤
	Control	30 (19/11)	63.28 ± 8.74		CT		
Xu (2021)	Trial	30 (16/14)	55.98 ± 6.52	NR	CT + MI	Guided by listening	②
	Control	30 (11/19)	56.72 ± 6.74		CT		
Deng (2017)	Trial	27	57.3 ± 10.2	Hemorrhage, Infarction	CT + MI, 30 min per session, once daily, a total of 8 weeks	Guided by listening	⑤
	Control	27			CT		
H. Zhang et al. (2017)	Trial	30	47–71	Hemorrhage, Infarction	CT + MI, 12–15 min per session, once daily, a total of 1 month	Guided by listening	④
	Control	30			CT		
Xu et al. (2017)	Trial	30 (25/5)	56.5 ± 11.8	Hemorrhage, infarction	CT + MI, 30 min per session, three sessions per day, a total of 6 weeks	Guided by listening	③
	Control	30 (26/4)	58.3 ± 11.2		CT		
Cao et al. (2014)	Trial	103 (85/18)	50.5 ± 11.8	Hemorrhage, Infarction	CT + MI, 30 min per session, once daily, a total of 6 weeks	Guided by listening	①⑤⑥
	Control	103 (86/17)	50.2 ± 11.6		CT		
Zhou (2020)	Trial	52 (30/22)	56.32 ± 6.87	Hemorrhage, infarction	CT + MI, 25–30 min per session, three sessions per day, a total of 6 weeks	Guided by listening	②
	Control	52 (28/24)	56.11 ± 6.76		CT		
Yang et al. (2023)	Trial	32(19/13)	58.32 ± 4.33	Infarction	CT + MI, 20 min per session, once daily, five sessions per week, a total of 3 weeks	Guided by listening and watching	①③
	Control	32(21/11)	59.11 ± 3.64		CT		
Liu et al. (2018)	Trial	50 (29/11)	56.17 ± 12.06	NR	CT+MI, 30 min per session, three sessions per day, a total of 6 weeks	Guided by listening and watching	②③
	Control	50 (28/12)	57.62 ± 10.42		CT		

*Note*: Outcomes (① Water swallowing test, ②Standardized swallowing assessment; ③ Swallowing quality of life; ④ Clinical effectiveness rate; ⑤ Complications; ⑥ Nasogastric tube removal rate).

Abbreviations: CT, conventional therapy; F, female; M, male; MI, motor imagery therapy. NR, not reported.

### 3.3 Risk of Bias of Included Studies

**FIGURE 2:**
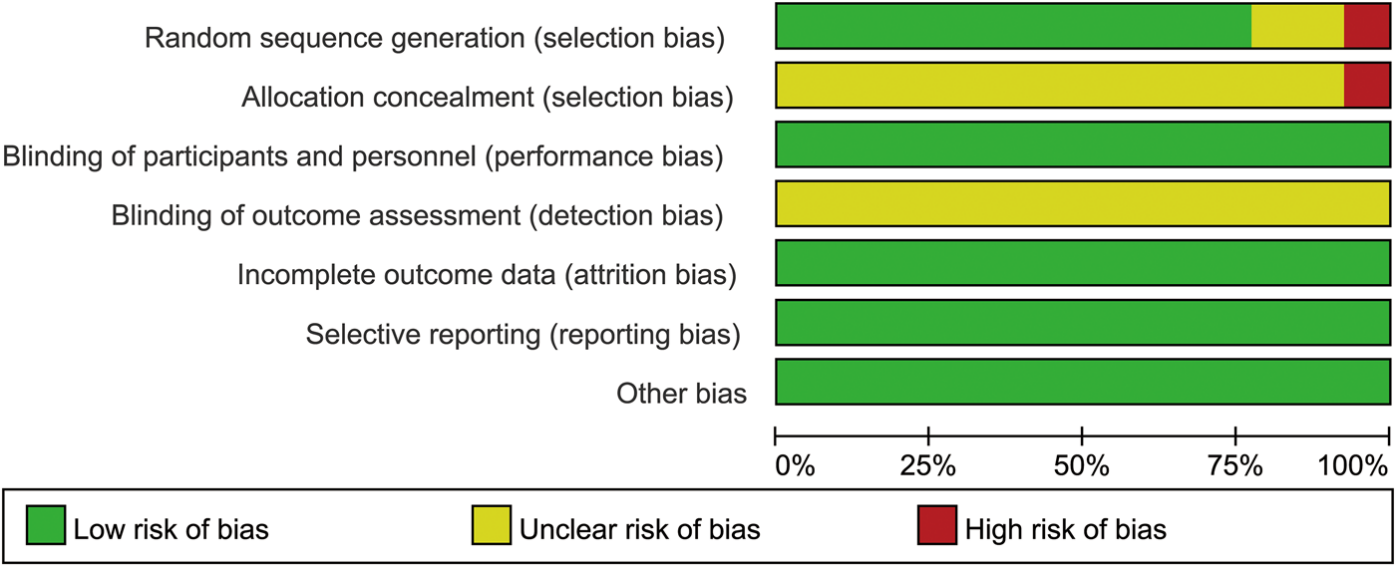
Risk of bias summary in the included studies.

**FIGURE 3:**
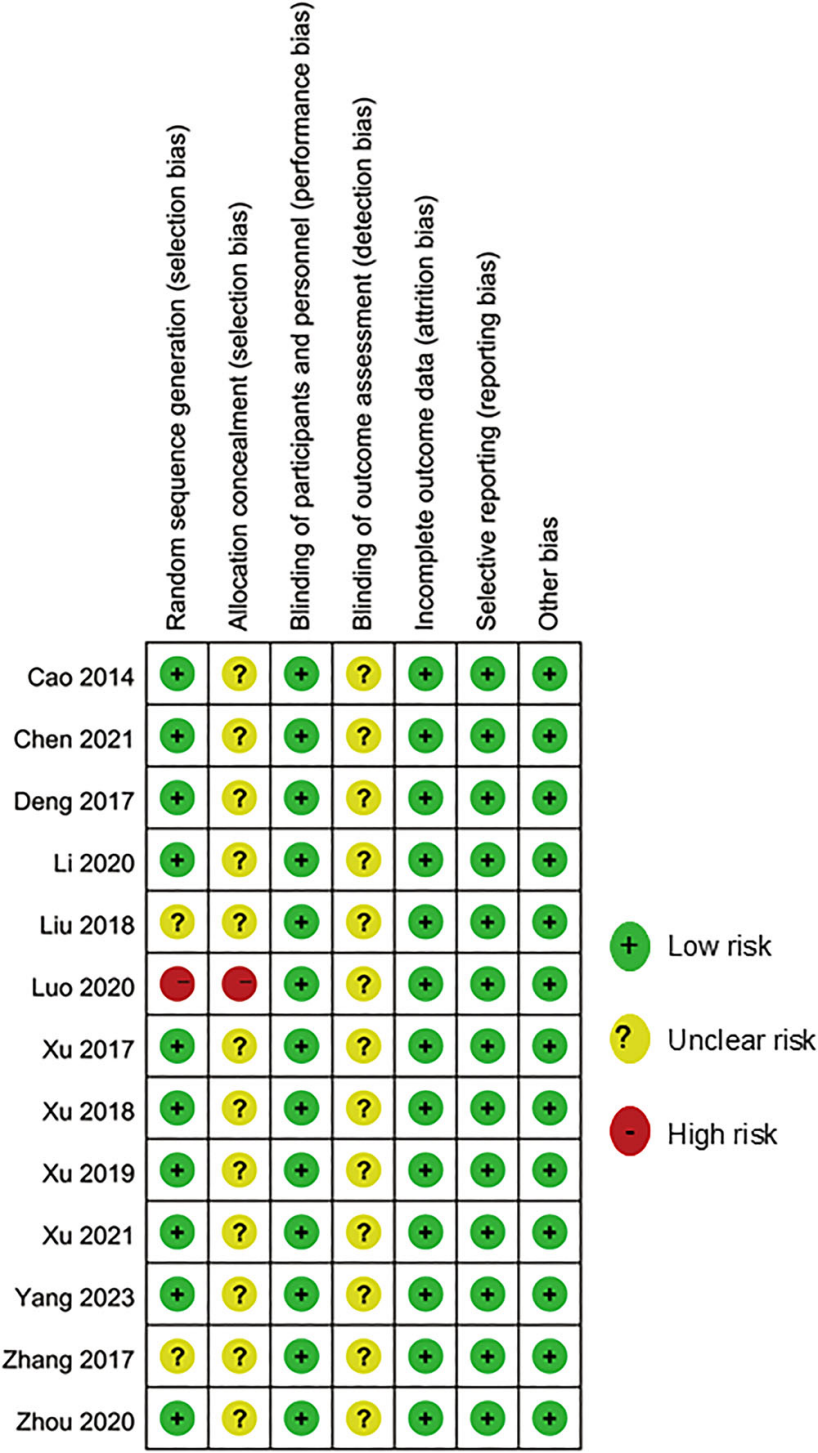
Methodological quality of the included studies.

Figures 2 and 3 show the risk of bias and the summary of methodological quality of the included articles, respectively. Among these included 13 studies; 1 study mistakenly used a grouping and allocation method based on diagnostic order (Luo et al. 2020), leading to selection bias. The other 12 studies reported using randomization, with 10 specifying the use of a random number table, lottery method, or grouping software for generating the random sequence. Regarding the allocation concealment, 12 studies lacked the relative details, resulting in an unclear risk of bias. Given the nature of MI intervention, blinding of patients was difficult for study design; this was an inherent issue in this type of research (R. Li et al. 2022). Thus, all the included studies were judged as “low risk” for performance bias. None of the studies mentioned blinding of the outcome measurements, which resulted in an increased risk of detection bias. All the included trials have complete outcome data, and none of the studies selectively reported outcomes. No evidence was found that there was other bias existing.

### 3.4 Water Swallowing Test

**FIGURE 4:**
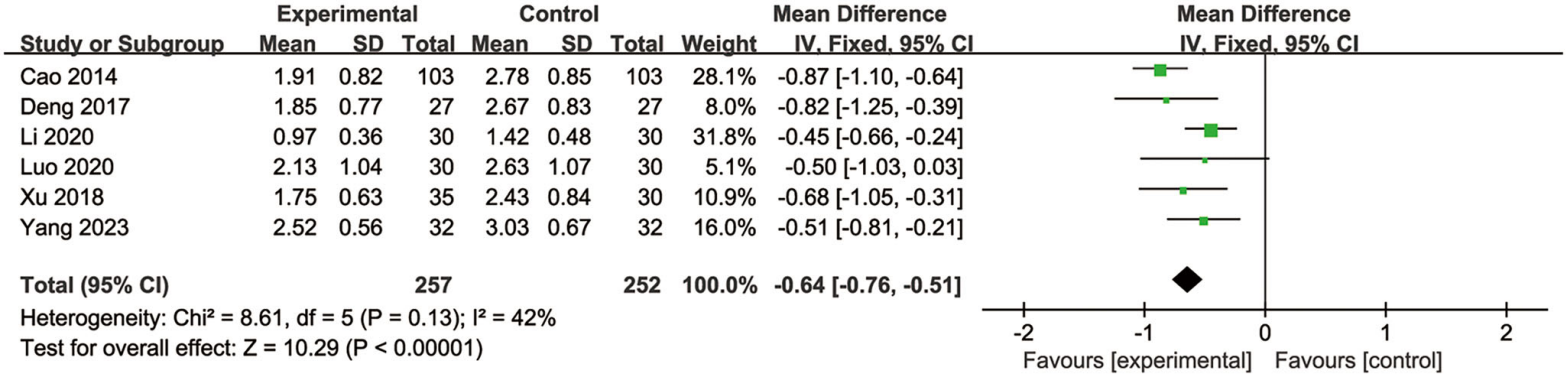
Forest plot comparing the water swallowing test between the two groups.

Six studies reported the results using the WST (Cao et al. 2014; Deng 2017; G. Li et al. 2020; Luo et al. 2020; Xu et al. 2018; Yang et al. 2023). The heterogeneity test results indicated *p* = 0.13 and *I* ^2^ = 42% (below the 50% threshold), suggesting no significant heterogeneity among the studies. Consequently, a fixed‐effect model was used for meta‐analysis. The forest plot's results showed a substantial effect of MI supplementation on decreasing WST score (MD = −0.64; 95% CI: −0.76, −0.51; *p* < 0.00001) (Figure 4).

### 3.5 Standardized Swallowing Assessment

**FIGURE 5:**
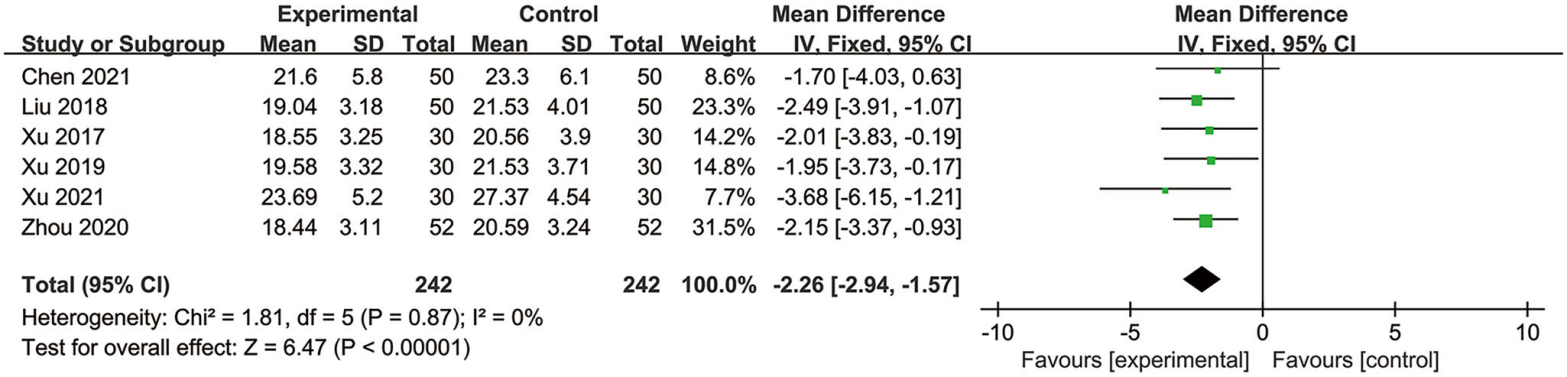
Forest plot comparing the standardized swallowing assessment between the two groups.

Six studies reported the SSA (Chen 2021; J. Liu et al. 2018; Xu 2021; Xu et al. 2017; Xu et al. 2019; Zhou 2020). None of the between‐study heterogeneity was detected (*p* = 0.87, *I* ^2^ = 0% < 50%); thus, a fixed effect model was employed. The pooled findings indicated that the SSA score was considerably lowered by MI therapy (MD = −2.26; 95% CI: −2.94, −1.57; *p* < 0.00001) (Figure 5).

### 3.6 Swallowing Quality of Life

**FIGURE 6:**
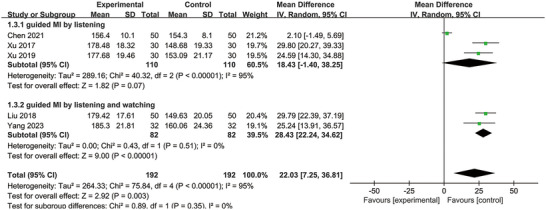
Forest plot comparing the swallowing quality of life between the two groups.

Five studies reported the SWAL‐QOL (Chen 2021; J. Liu et al. 2018; Xu et al. 2017; Xu et al. 2019; Yang et al. 2023). The heterogeneity test indicated significant variability among studies, with *p* < 0.00001 and *I* ^2^ = 95%, which exceeded the 50% threshold. A random effect model indicated that MI supplementation enhanced the SWAL‐QOL scores after treatment (MD = 22.03; 95% CI: 7.25, 36.81; *p* = 0.003) (Figure 6). In addition, sensitivity analysis showed that after excluding Chen's study (Chen 2021), the *I* ^2^ value dropped from 95% to 0%, while the overall effect remained stable, indicating that Chen's study was the primary source of heterogeneity.

In these studies, for guiding MI treatment, auditory modality (verbal cues) and visual modality (action demonstration) were adopted. Therefore, we performed subgroup analysis according to the guided MI modality. Subgroup results further showed that guided MI by both listening and watching had a positive effect size (MD = 28.43; 95% CI: 22.24, 34.62; *p* < 0.00001); however, guided MI only by listening had no significantly positive effect size (MD = 18.43; 95% CI: −1.4, 38.25; *p *= 0.07), as present in Figure 6.

### 3.7 Clinical Efficacy

**FIGURE 7:**
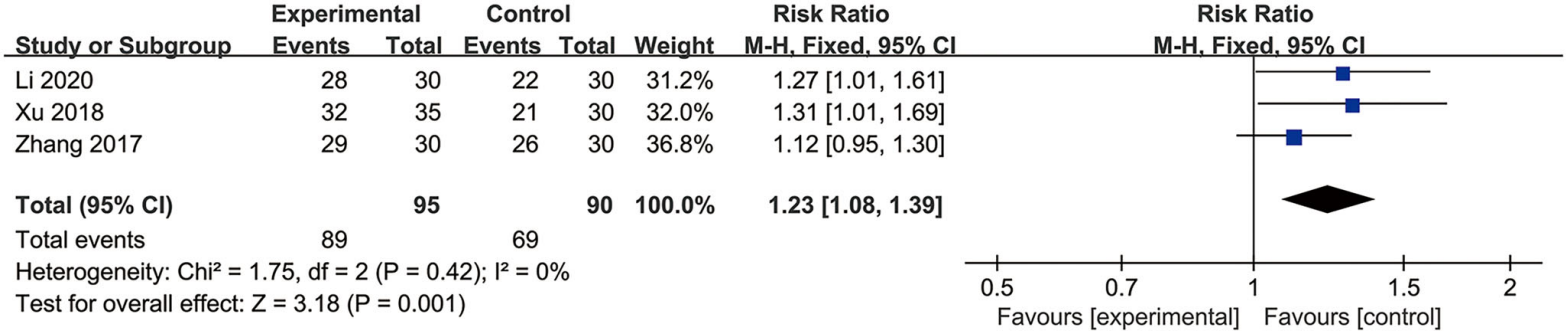
Forest plot comparing clinical efficacy between two groups.

A total of three studies reported the clinical effectiveness rate, which was evaluated using WST (G. Li et al. 2020; Xu et al. 2018) or the dysphagia rating scale (H. Zhang and Ma 2017). According to the results of the WST or dysphagia rating scale, clinical efficacy was defined as either cured cases or effective cases, whereas cases showing no improvement were classified as ineffective. A meaningful effect of MI treatment was observed on clinical efficacy from the pooled result (RR = 1.23; 95% CI: 1.08, 1.39; *p* = 0.001) (Figure 7). No statistically significant heterogeneity was observed. (*p* = 0.42, *I* ^2^ = 0% < 50%).

### 3.8 Complications

**FIGURE 8:**
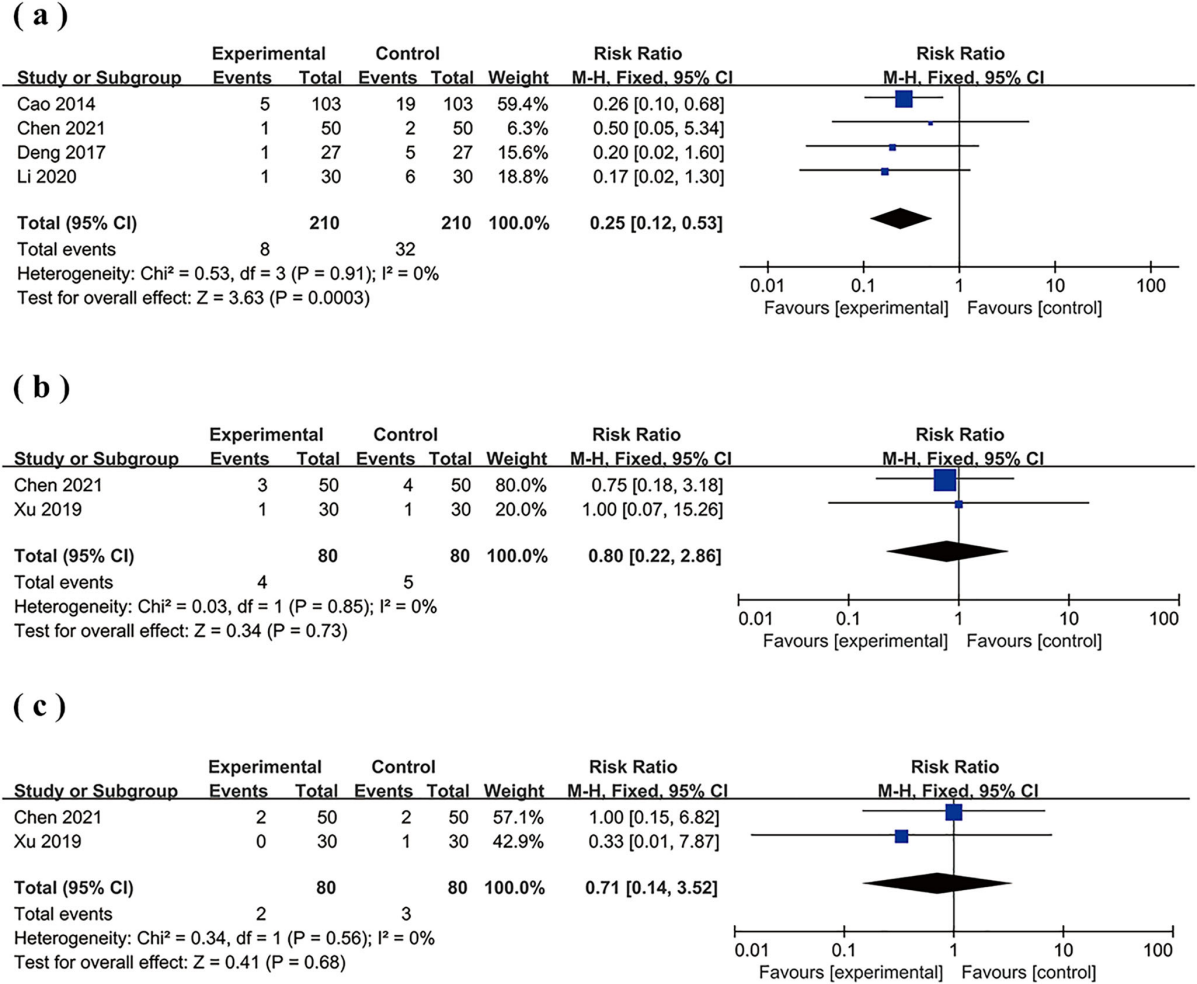
Forest plot comparing the incidence of complications between the two groups. (a) Aspiration pneumonia; (b) aspiration; (c) malnutrition.

A total of five studies reported complications (Cao et al. 2014; Chen 2021; Deng 2017; G. Li et al. 2020; Xu et al. 2019). Four of these studies reported on aspiration pneumonia (Cao et al. 2014; Chen 2021; Deng 2017; G. Li et al. 2020), two studies reported on aspiration and malnutrition (Chen 2021; Xu et al. 2019), and one study reported on dehydration (Chen 2021). The pooled results revealed that MI intervention led to a significant reduction in the incidence rate of aspiration pneumonia (RR = 0.25; 95% CI: 0.12, 0.53; *p* = 0.0003. *I* ^2^ = 0%) (Figure 8a). However, results did not show any meaningful effect of MI supplementation on aspiration (RR = 0.8; 95% CI: 0.22, 2.86; *p* = 0.73. *I* ^2^ = 0%) (Figure 8b) or malnutrition (RR = 0.71; 95% CI: 0.14, 3.52; *p* = 0.68. *I* ^2^ = 0%) (Figure 8c). Additionally, regarding dehydration, three cases were observed in the trial group, while two cases were found in the control group. Table 2 shows the complications reported in the involved studies.

**TABLE 2:** Summary of complications of the involved studies.

Study ID	Group	Number	Number of patients with complications (*n*)
G. Li et al. (2020)	Trial	30	aspiration pneumonia (*n* = 1)
	Control	30	aspiration pneumonia (*n* = 6)
Chen (2021)	Trial	50	aspiration pneumonia (*n* = 1); aspiration (*n* = 3); malnutrition (*n* = 2); dehydration (*n* = 3)
	Control	50	aspiration pneumonia (*n* = 2); aspiration (*n* = 4); malnutrition (*n* = 2); dehydration (*n* = 2)
Xu et al. (2019)	Trial	30	aspiration (*n* = 1)
	Control	30	aspiration (*n* = 1); malnutrition (*n* = 1)
Deng 2017	Trial	27	aspiration pneumonia (*n* = 1)
	Control	27	aspiration pneumonia (*n* = 5)
Cao et al. 2014	Trial	103	aspiration pneumonia (*n* = 5)
	Control	103	aspiration pneumonia (*n* = 19)

### 3.9 Nasogastric Tube Removal Rate

**FIGURE 9:**
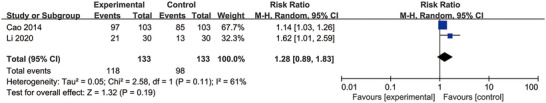
Forest plot comparing the proportion of nasogastric tube removal between the two groups.

Two studies recorded the nasogastric tube placement among patients (Cao et al. 2014; G. Li et al. 2020). In Li's study, 21 patients (70%) met the criteria for nasogastric tube removal in the trial group, which was greater than that in the control group (*n* = 13, 43.33%). In the other study, the rate of nasogastric tube removal was reported as follows: 97 patients in the trial group successfully removed the nasogastric tube, accounting for 94.17% of the number of participants, while in the control group, this proportion was 82.52%. Due to the high heterogeneity, the random effect model was used for meta‐analysis; however, the pooled results failed to show that the proportion of patients meeting the criteria for nasogastric tube removal in the trial group was significantly greater than that in the control group (RR = 1.28; 95% CI: 0.89, 1.83; *p* = 0.19: *I* ^2^ = 61%) (Figure 9).

### 3.10 Bootstrapped Meta‐Analysis

**FIGURE 10:**
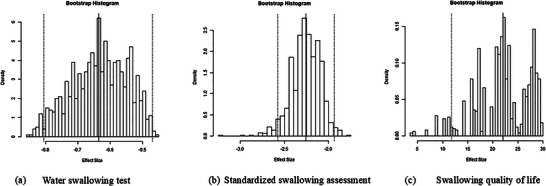
The histograms of bootstrapped meta‐analysis using 1000 replicates. (a) Bootstrap histogram for water swallowing test; (b) bootstrap histogram for standardized swallowing assessment; (c) bootstrap histogram for swallowing quality of life.

The results of a bootstrapped meta‐analysis following 1000 bootstrap replicates (Figure 10; Table S3) validated the accuracy of the actual meta‐analysis obtained from RCTs. The effect estimates over the replicates remained significant for the primary outcomes evaluated: WST −0.64 (95% CI: −0.81, −0.47); SSA −2.26 (95% CI: −2.58, −1.94); SWAL‐QOL 22.03 (95% CI: 11.75, 32.21). Bootstrapping meta‐analysis was not feasible for secondary outcomes (including clinical efficacy, complications, and nasogastric tube removal rate) due to the few available RCTs (less than five included studies) (Alonso et al. 2025).

## 4 Discussion

In the realm of stroke rehabilitation, MI has gained increasing reputation among therapists and clinicians in recent years due to its ability to facilitate neuroplasticity recovery and restore physical functions poststroke (R.Q. Li et al. 2017). This systematic review and meta‐analysis, which included 13 RCTs with 1053 patients, evaluated the existing evidence on the efficacy of MI as an add‐on therapy for people experiencing poststroke dysphagia.

Overall, the findings of this study demonstrated that compared with conventional therapy alone, MI, as a complementary approach, has a positive effect on improving swallowing function, swallowing‐related quality of life, and clinical effectiveness rate, while also reducing complications such as aspiration pneumonia—a major contributor to mortality, morbidity, and institutionalization in stroke patients, accounting for approximately 35% of poststroke mortality (Feng et al. 2019). This meta‐analysis assessed the WST, SSA, SWAL‐QOL, clinical efficacy, complications, and feeding via nasogastric tube. Based on our findings, MI intervention was found to positively influence WST, SSA, SWAL‐QOL, clinical efficacy, and incidence of aspiration pneumonia when compared with conventional treatment used alone. Given the limited number of studies (a mere two) included for outcomes such as aspiration, malnutrition, and proportion of nasogastric tube removal, no statistically significant differences were detected between the groups.

The pooled result of SWAL‐QOL exhibited significant heterogeneity. Sensitivity analysis of SWAL‐QOL suggested that Chen's study (Chen 2021), which exclusively enrolled hemorrhagic stroke patients and adopted a three‐arm parallel RCT design (unlike the other two‐group parallel RCTs), might be the source of heterogeneity due to the clinical differences and methodological inconsistencies. Further subgroup analysis of SWAL‐QOL was conducted based on the type of MI‐guided modality used. Two modalities were identified in included RCTs: (1) MI guided by a combination of auditory instruction and action observation, and (2) MI guided by auditory instruction alone. Specifically, in the first modality, patients mentally simulated the swallowing process guided by watching swallowing‐related actions and listening to swallowing‐related instruction. In contrast, the second modality involved mental simulation of swallowing guided solely by auditory instruction. Subgroup analysis demonstrated that the combined visual–auditory modality significantly improved SWAL‐QOL scores, whereas the auditory‐only modality showed no significant effect.

Regarding the quality of the included studies, the Cochrane tool revealed that all studies were rated as having an unclear risk of bias for outcome assessor blinding. In the study by Luo et al. (2020), both random sequence generation and allocation concealment were improperly implemented, resulting in a high risk of selection bias. One study failed to describe the random sequence generation process and was consequently classified as having an uncertain risk of bias. Additionally, 12 studies did not report allocation concealment procedures. According to the Cochrane bias assessment tool, our analysis also demonstrated that all the included studies presented a low risk of performance bias, attrition bias, reporting bias, and other potential biases. Furthermore, 10 studies correctly implemented random sequence generation using appropriate methods.

Regarding the MI protocol used in the included studies, apparent differences in exposure times of MI training protocols were observed. Most of the included studies implemented MI interventions lasting 3–8 weeks, with training frequency varying significantly from 12 to 90 min per day. Because of heterogeneous patient samples, such as the variability of time since stroke onset and lesion characteristics in different patient samples, this review cannot answer questions concerning the optimal timing of MI intervention as well as structured intervention frequency and duration. Therefore, future research should further investigate the best practices in MI intervention for patient benefit. Although the inclusion criteria of this meta‐analysis concerned no restriction on the type of MI (kinesthetic imagery and visual imagery), all the included studies adopted kinesthetic imagery. During kinesthetic imagery, patients experienced movement sensations and visualized themselves fully executing the swallowing action from an internal perspective. Unlike kinesthetic imagery, during visual imagery, subjects feel as though they see themselves or another person performing the movement from an external perspective (Shen et al. 2025). The findings of Stinear et al. (2006) indicate that kinesthetic imagery is more effective for motor learning than visual imagery. Further neuroimaging research revealed that the underlying mechanism for the superior effect of kinesthetic imagery can be linked to the activation of the somatosensory cortex (Decety and Grèzes 2006). A study involving Parkinson's patients demonstrated that kinesthetic imagery exhibits efficacy in improving motor function, which was correlated with a significant increase in the amplitude of the contingent negative variation (CNV) late component—an electrophysiological reflection of primary motor cortex activity. In contrast, visual imagery showed no effect on the CNV late component (Lim et al. 2006). Another study indicated that visual imagery primarily enhances cognitive ability rather than restoring motor function in stroke patients (K. P. Liu et al. 2004). Moreover, visual imagery strongly relies on continuous visual stimulation throughout the entire intervention process (Shen et al. 2025). Consequently, complementary kinesthetic imagery is widely used in motor function rehabilitation due to its superior efficacy and easier implementation compared to visual imagery.

Since swallowing MI is difficult to perform smoothly without appropriate guidance, all MI tasks in the included studies were guided by auditory instruction or by a combination of auditory instruction and action observation. As mentioned above, subgroup analysis of SWAL‐QOL was performed based on guidance modality. Notably, the pooled result supported the significant therapeutic benefit for the swallowing‐related quality of life of MI guided by a combination of auditory instruction and action observation rather than by auditory instruction only. This finding may be explained by the fact that when patients receive cognitive induction through swallowing‐related action demonstration and listening instruction, the dual sensory pathways (visual and auditory senses) are used to perceive and judge the food characters, with central nervous system synthesis of these signals, which can reinforce the vividness of the mental imagery and subsequent MI treatment effectiveness (Wang et al. 2024). Therefore, based on this finding, future research should continue to investigate the differences among the guidance modalities to further facilitate the establishment of a standardized protocol for MI training and ultimately to benefit patients.

Beyond the MI intervention, variations in control intervention types were noted. The conventional therapy adopted in the control groups of included studies was based on an integrated approach incorporating dietary intervention, behavioral therapy, neurostimulation, ice stimulation, and so forth. However, these differences do not compromise the comparability of included studies regarding treatment efficacy for poststroke dysphagia. All included RCTs compared MI combined with conventional therapy to conventional therapy alone, and the conventional therapy in the experimental and control groups is identical. Consequently, the pooled findings from this meta‐analysis provide a valid representation of MI's therapeutic efficacy in poststroke dysphagia rehabilitation.

Regarding participants, all included RCTs excluded patients with severe cognitive impairment who might exhibit chaotic MI execution. Although stroke patients exhibit varying degrees of neurological impairments, the implementation of MI therapy requires adequate MI ability. Consequently, this intervention is seldom applied in rehabilitating individuals with severe cognitive dysfunction (Sharma et al. 2009). According to the reported data in the included studies, patients ranged from acute to chronic phases, with a predominance was in the acute and subacute phases. Although evidence indicates that the timely implementation of MI is essential for functional recovery in stroke patients, studies also demonstrate that MI remains effective in individuals > 1 year poststroke. This is because goal‐directed mental simulation not only strengthens synaptic connections but also enhances compensatory neural networks (Huda Arif et al. 2025; Kang et al. 2021; Page et al. 2005).

Overall, to the best of our knowledge, this represents the first systematic review and meta‐analysis investigating the efficacy of MI as an adjunctive therapy for poststroke dysphagia. Based on the existing evidence, our findings address the research question by demonstrating the potential of MI as a promising add‐on intervention, with benefits for improving swallowing function, enhancing quality of life, increasing clinical effectiveness, and reducing serious complications in stroke survivors.

### 4.1 Strengths and Limitations

Given that the systematic review on the role of MI in poststroke dysphagia treatment remains a neglected area, this meta‐analysis was undertaken to specifically investigate the impact of MI as a supplementary treatment for managing dysphagia following a stroke. It has been revealed that MI intervention has the potential to improve WST, SSA, SWAL‐QOL, clinical efficacy, and incidence of aspiration pneumonia among stroke patients with swallowing dysfunction. Moreover, the robustness and accuracy of conventional pooling results from the primary outcomes study (regarding WST, SSA, and SWAL‐QOL) were further confirmed by bootstrapping meta‐analysis. Nonetheless, it is important to acknowledge the study's limitations. First, there was heterogeneity between the studies selected in terms of MI‐guided modality, intervention frequency and duration, and time since stroke onset. The difference in type of stroke may have led to substantial statistical heterogeneity in the meta‐analysis of SWAL‐QOL. We did not conduct publication bias analysis, as the test is less reliable with fewer than 10 included studies. In addition, one RCT included in this study holds certain methodological flaws, such as a high risk of bias in terms of random sequence generation and allocation concealment, which may affect the credibility of results. Third, all included RCTs were conducted in China, which may limit the generalization of our findings. However, this does not mean that no clinical MI studies focusing on dysphagia rehabilitation were reported in other languages. One RCT applied MI training in dysphagia patients with Wallenberg syndrome (Wang et al. 2024), and another study used a multiple case study to compare the effect of motor execution and MI of swallowing in dysphagia patients with cerebral lesions (Kober et al. 2015). We excluded these studies during study selection. Thus, broader and higher‐quality RCTs are warranted to support our conclusion. Lastly, the reliance on assessment scales in most studies introduces potential subjective bias into the evaluation process; hence, more objective evaluation indicators are needed, such as incorporating neuroimaging techniques, biochemical methods, etc., to assess the outcome.

## 5 Conclusion

In the present study, we provided the first meta‐analytic evidence that the add‐on MI intervention is beneficial for the management of swallowing dysfunction following stroke. However, given the inherent limitations of the reviewed studies, there is a need for additional high‐quality, large‐scale, multicenter RCTs to provide more conclusive evidence supporting the clinical application of MI intervention for poststroke dysphagia management. It would also be worthwhile to continually explore the optimal training protocol of MI regarding the intervention frequency and duration, types of strokes, and guidance modalities.

## Author Contributions

**Yunlu Liu**: conceptualization, data curation, writing – original draft. **Yang Wang**: conceptualization, writing – review and editing, funding acquisition. **Xinyu Zhao**: methodology, software, formal analysis, investigation, data curation. **Peiqun Hu**: investigation. **Yushi Hu**: funding acquisition, supervision.

## Ethics Statement

The authors have nothing to report.

## Conflicts of Interest

The authors declare no conflicts of interest.

## Peer Review

The peer review history for this article is available at https://publons.com/publon/10.1002/brb3.70826

## Supporting information




**Supplementary Material**: brb370826‐sup‐0001‐SuppMatt.docx

## Data Availability

All data supporting the findings of this study are available within the paper and its Supporting Information.
